# Influence of Different Age Cutoff Points on the Prediction of Prognosis of Cancer Patients Receiving ICIs and Potential Mechanistic Exploration

**DOI:** 10.3389/fonc.2021.670927

**Published:** 2021-06-23

**Authors:** Rui Guan, Qiong Lyu, Anqi Lin, Junyi Liang, Weimin Ding, Manming Cao, Peng Luo, Jian Zhang

**Affiliations:** Department of Oncology, Zhujiang Hospital, Southern Medical University, Guangzhou, China

**Keywords:** pan-cancer, ICI, age, predictive markers, prognosis

## Abstract

Age is a potential predictive marker for the prognosis of cancer patients treated with immune checkpoint inhibitors (ICIs), but the appropriate age cutoff point is still controversial. We aimed to explore the influence of different age cutoff points on the prediction of prognosis for patients receiving ICIs and explore the mechanism underlying the appropriate age cutoff point from the aspects of gene mutation and expression, immune cell infiltration and so on. We applied cutoff points of 50, 55, 60, 65, 70, and 75 years old to divide 1660 patients from the Memorial Sloan-Kettering Cancer Center (MSKCC) immunotherapy cohort into older and younger groups and performed survival analysis of the six subgroups. The results showed that older patients had better survival than younger patients in accordance with the cutoff point of 50 years old [median overall survival (OS) (95% CI): 13.0 (10.5-15.5) months *vs.* 20.0 (16.7-23.3) months; p=0.002; unadjusted hazard ratio (HR) (95% CI): 0.77 (0.65-0.91)], whereas no significant difference was observed with other cutoff points. Further analysis of The Cancer Genome Atlas (TCGA) database and the MSKCC immunotherapy cohort data showed that the tumor mutation burden (TMB), neoantigen load (NAL), DNA damage response and repair (DDR) pathway mutation status, mutation frequencies of most genes (except IDH1, BRAF and ATRX), the expression of most immune-related genes and the degree of infiltration of most immune cells (such as CD8+ T cells and M1 macrophages) were higher in the elderly group (aged ≥50 years).

## Introduction

In the 21st century, cancer is expected to become the leading cause of death and the leading obstacle to increased life expectancy in countries around the world. According to the 2020 Cancer Statistics report, there were approximately 19.3 million new cancer cases and 10.0 million cancer deaths worldwide (1). The most commonly diagnosed cancer types are female breast cancer (11.7% of all cases), lung cancer (11.4%), colorectal cancer (10.0%) and prostate cancer (7.3%) (1). The leading cause of cancer-related death is lung cancer (18% of all cancer-related deaths), followed by colorectal cancer (9.4%), liver cancer (8.3%) and stomach cancer (7.7%) (1). With the increase in the screening rate and advancements in diagnosis and treatment technology, the prognosis of cancer patients has been greatly improved. However, it was reported that the overall 5-year relative survival rate of all cancers diagnosed between 2009 and 2015 was only 67%, with pancreatic cancer (9%), liver cancer (18%), lung cancer (19%) and esophageal cancer (20%) having the lowest 5-year survival rates (2).

With the in-depth understanding of the tumor microenvironment, immunotherapy has become a new standard treatment strategy in addition to traditional therapies such as surgery, radiotherapy and chemotherapy. Immune checkpoint inhibitors (ICIs) have made a breakthrough in clinical treatment. ICIs mainly include CTLA-4 inhibitors and PD1/PD-L1 inhibitors, which have antitumor effects by blocking inhibitory receptors on T cells and reactivating T cells. Currently, ICIs are approved for melanoma, lung cancer, bladder cancer, kidney cancer, lymphoma and so on and have significantly improved the survival rate of patients with many kinds of cancer (3). Unfortunately, only a small subset of patients can benefit from these drugs. For example, the objective response rates (ORRs) of pembrolizumab or nivolumab in the first-line treatment of melanoma and the second-line treatment of non-small-cell lung cancer (NSCLC) are 40-45% and 20%, respectively (4–6). Therefore, it is necessary to identify biomarkers for predicting the efficacy of ICIs to screen the patients who may benefit from ICIs.

Many molecular biological markers related to the prognosis of ICIs have been gradually established. Changes in the intrinsic factors of tumor cells, such as increased tumor mutation burden (TMB) and increased expression of PD-L1, are usually associated with increased clinical benefits in patients (3). Specific gene mutations can also affect the response to ICIs. For example, patients with LRP1B-mutant cancer showed longer progression-free survival (PFS) and overall survival (OS) than wild-type patients when treated with ICIs (7). In addition, the degree of infiltration of all kinds of immune cells and the expression of various cytokines in the tumor microenvironment also affects the results of immunotherapy (3).

In addition to tumor-related factors, host characteristics such as age and sex may also be predictive markers for the efficacy of ICIs (3). Among them, age, as one of the most important clinical features of cancer patients, has shown a positive impact on prognosis in some studies. For example, studies by Kugel et al. showed that melanoma patients aged ≥60 years responded better to PD1 inhibitors than younger patients (8). Another meta-analysis of melanoma showed that patients over 75 years old were more likely to benefit from ICIs (9). For pan-cancer patients, Wu et al. found that the prognosis of older patients (≥65 years old) using ICIs was better than that of younger patients (<65 years old) (10). However, some studies have revealed the adverse effect of age on prognosis. For example, Li et al. showed that older cancer patients tend to have better OS and PFS (11). In addition, some studies have shown that age is not an independent predictor of prognosis in cancer patients treated with ICIs. For example, Sun et al. revealed that there was no significant difference in the OS of patients with NSCLC using PD-1/PD-L1 inhibitors between the young group (< 65 years old) and the old group (≥65 years old) (12). At the cutoff point of 70 years old, the PFS and OS of patients treated with ICIs in the melanoma, NSCLC and pan-cancer datasets were similar (13–15). Another analysis of renal cell carcinoma (RCC) and urothelial carcinoma (UC) as a whole or in subgroups showed that older patients over 75 years old had disease control rates (DCRs) comparable to those of younger patients treated with ICIs (16). The controversial results mentioned above may be related to the cancer type, sample size, and age cutoff point. At present, the selection of the age cutoff point is still controversial, leading to discrepancies in the age cutoff points presented in different studies. In addition, we found that for a single cancer type, such as melanoma, or for a collection of cancer types, the selection of different age cutoff points is likely to affect the predictive impact of age on the prognosis of patients treated with ICIs. Therefore, the selection of the appropriate age cutoff point is still worthy of further exploration.

In summary, this study aims to explore the appropriate age cutoff point for ICI efficacy prediction. Considering that the effect of age on the prognosis of patients using ICIs may be impacted by various age-related negative/positive predictive markers, we intended to use the data obtained from the database and literature to try to reveal the biomarkers related to age from the perspective of gene mutations, gene expression levels, immune cell infiltration and related pathways.

## Materials and Methods

### Data Acquisition

The clinical phenotypic information of The Cancer Genome Atlas (TCGA) sample was downloaded from UCSC Xena Browser. The TCGA pan-cancer gene mutation data (Mutations-mc3.v0.2.8.PUBLIC.maf.gz) and gene expression data [RNA (Final) -EBPlusPlusAdjustPANCAN_IlluminaHiSeq_RNASeqV2.geneExp.tsv] were downloaded from NCI’s Genomic Data Commons (GDC) (https://gdc.cancer.gov/about-data/publications/pancanatlas). In addition, clinical data and mutation data of 1661 pan-cancer patients receiving ICI treatment [namely, Memorial Sloan-Kettering Cancer Center (MSKCC) immunotherapy cohort] were downloaded from cBioPortal (17), and the mutation data were obtained from targeted next-generation sequencing (MSK-IMPACT) (18). Finally, the data needed for the analysis of neoantigen load (NAL) and immune cell infiltration and the list of immune-related genes were obtained from published literature (19).

### Analysis of TMB, Mutation Numbers in the DNA Damage Response and Repair Pathway and Gene Mutations

As in the other literature, a nonsynonymous mutation from the TCGA database was used as the raw mutation count, and it was divided by 38 MB to quantify TMB (20). In the MSKCC immunotherapy cohort, TMB was equivalent to the total number of nonsynonymous mutations.

The DDR pathway gene set was derived from the Molecular Signatures Database (MSigDB) of the Broad Institute (21). It was used to evaluate the number of nonsynonymous mutations in the DDR pathway in the MSKCC immunotherapy cohort and the TCGA database.

Since the number of samples in the elderly group in the TCGA database was much larger than that in the young group, we randomly selected samples from the elderly group (seed=2107) to match the number of samples from the young group and carried out subsequent gene mutation analysis. The R package ‘ComplexHeatmap’ (22) was used to visualize genes whose mutation rate in the elderly group was greater than 10% or whose mutation rate in the young group was higher than that in the elderly group in the TCGA database.

### Analysis of Immune Gene Expression and Immune Cell Infiltration

CIBERSORT was run using the LM22 signature and 1000 permutations to estimate the relative fractions of 22 immune cell types in the TCGA samples described by Thorsson et al. We obtained the relative abundance of immune cell infiltration in TCGA samples from the supplementary data published by Thorsson (19), subsequently explored the difference in immune cell infiltration in different age groups, and analyzed the correlation between the abundance of immune cell infiltration and the differentially expressed pathways.

With reference to immune-related genes and their functional classification set provided by Thorsson et al. (19), mRNA expression levels of immune-related genes quantified as log2 (transcripts per million [TPM]) were compared between different age groups in TCGA database.

### Analysis of the Correlation Between the Differentially Expressed Pathways and the Infiltration Scores of 22 Immune Cell Types

The gene expression data (fragments per kilobase of transcript per million mapped reads; FPKM) of the TCGA pan-cancer dataset downloaded from the GDC were converted into TPM format for subsequent analysis. Single-sample gene set enrichment analysis (ssGSEA) was performed on the expression data using the R package ‘gsva’ (23), and differential expression analysis of the pathway was completed by combining the use of the R package ‘limma’ (24), where an ad.p.value<0.01 in Reactome was considered to be significantly different. In the gene set variation analysis (GSVA), an ad.p.value<0.01 was considered statistically significant. In addition, we analyzed the correlation between the differentially enriched pathways and the infiltration scores of 22 immune cell types obtained by CIBERSORT analysis.

### Statistical Analysis

**Figure 1:**
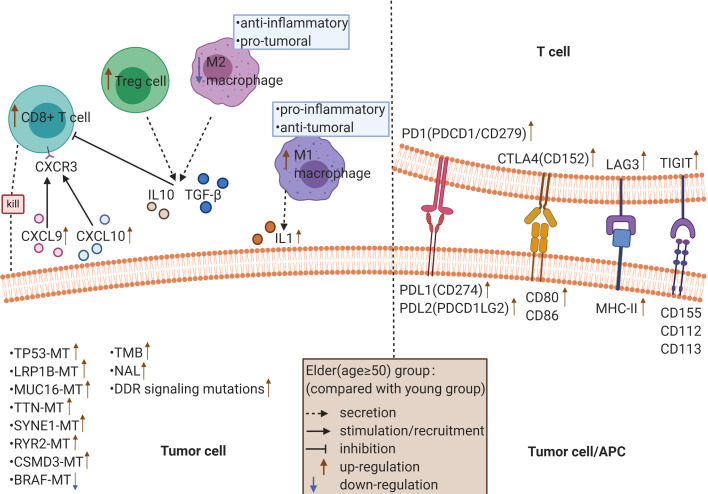
The possible mechanism underlying the improved efficacy and prognosis in older cancer patients (≥ 50 years old) receiving ICIs.

The R packages ‘survival’ and ‘survminer’ (25) were used for survival analysis to explore appropriate age cutoff points. The Mann-Whitney U test was used to compare the TMB, NAL, number of gene mutations in DDR pathways, expression levels of immune-related genes and infiltration scores of immune cells between the young group and the elderly group. Fisher’s exact test was used to compare the difference in gene mutations in the TCGA database between the young group and elderly group. P<0.05 was considered significantly different, and all statistical tests were bilateral. All statistical tests and visual analysis were performed in R software (version 3.6.1). In addition, the R package ‘ggpurb’ (26) was used to calculate statistical parameters in a visual boxplot. A mechanism diagram (**Figure 1**) was drawn using BioRender software.

## Results

### Effect of Different Age Cutoff Points on the Prognosis of Pan-Cancer Patients Receiving ICIs

Our study included 1660 patients (a patient whose exact age was unknown was excluded) from the MSKCC. The patients had non-small-cell lung cancer (n=349), melanoma (n=320), bladder cancer (n=215), RCC (n=151), head and neck cancer (n=139), esophagogastric cancer (n=126), glioma (n=117), colorectal cancer (n=110), breast cancer (n=44), skin cancer (n=1), or cancer of an unknown primary origin (n=88). The ages of the patients ranged from 15 to 90 years old. Of the patients enrolled, 1033 were male, 1036 received PD-1/PD-L1 inhibitors [PD-1/PD-L1 group], 99 received CTLA-4 inhibitors (CTLA-4 group), and 255 received a combination of PD-1/PD-L1 inhibitors and CTLA4 inhibitors (combo group).

**Figure 2:**
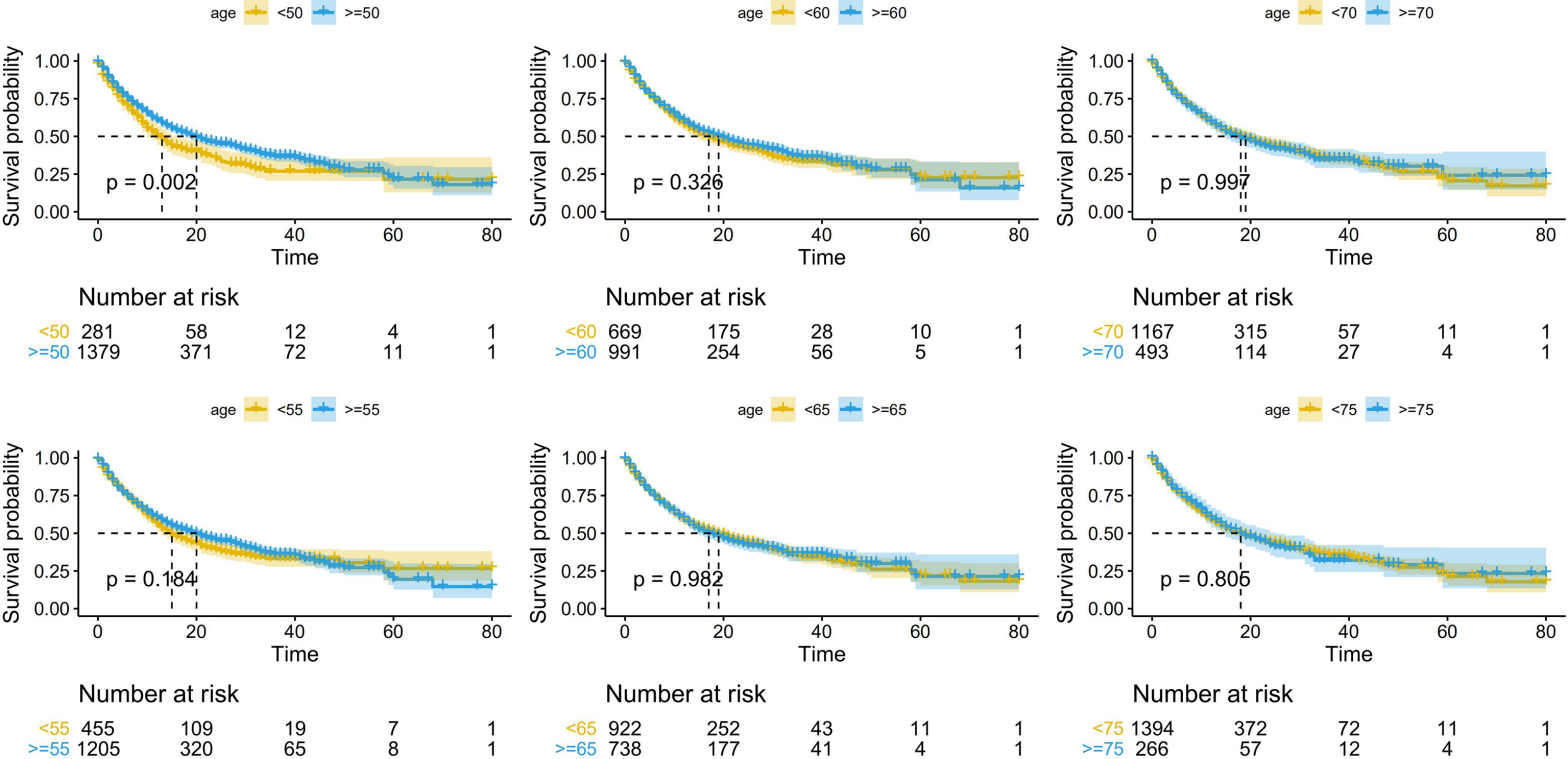
Kaplan-Meier curves depicting overall survival (OS; in months) according to different age cutoff points in patients receiving immune checkpoint inhibitors (ICIs).

Using cutoff points of 50, 55, 60, 65, 70, and 75 years old, we divided patients into older and younger groups and performed survival analysis of the six subgroups with R software (survival package and survminer package). The results showed that older patients had better survival than younger patients in accordance with the cutoff point of 50 years old [median OS (95% CI): 13.0 (10.5-15.5) months *vs.* 20.0 (16.7-23.3) months; p=0.002; unadjusted hazard ratio (HR) (95% CI): 0.77 (0.65-0.91)], whereas no significant difference was observed with other cutoff points (**Figure 2**). In addition, we designed 18 subgroups that were randomly combined with three different treatment groups and the six age cutoff points mentioned above (**Figures S1**–**S3**). The results revealed that patients aged ≥50 years in the PD-1/PD-L1 group [median OS (95% CI): 11.0 (8.5-13.5) months *vs.* 15.0 (13.1-16.9) months; p=0.027; unadjusted HR (95% CI): 0.80 (0.65-0.98)], patients aged ≥50 years in the combo group [median OS (95% CI): 14.0 (5.6-22.4) months *vs.* 49.0 (37.6-60.4) months; p<0.001; unadjusted HR (95% CI): 0.36 (0.23-0.54)], and patients aged ≥55 years in the combo group (median OS (95% CI): 21.0 (15.1-26.9) months *vs.* 46.0 (not reached) months; p<0.001; unadjusted HR (95% CI): 0.47 (0.31–0.70)] survived longer than younger patients. No significant difference was observed in the remaining subgroups.

In summary, 50 years old may be the appropriate age cutoff point related to the efficacy of ICIs. Below, patients were divided into an elderly group and a young group, with 50 years old as the cutoff, to explore the biomarkers hidden behind age.

### Analysis of TCGA Gene Mutation and Gene Expression Data Between the Elderly Group and the Young Group

**Figure 3:**
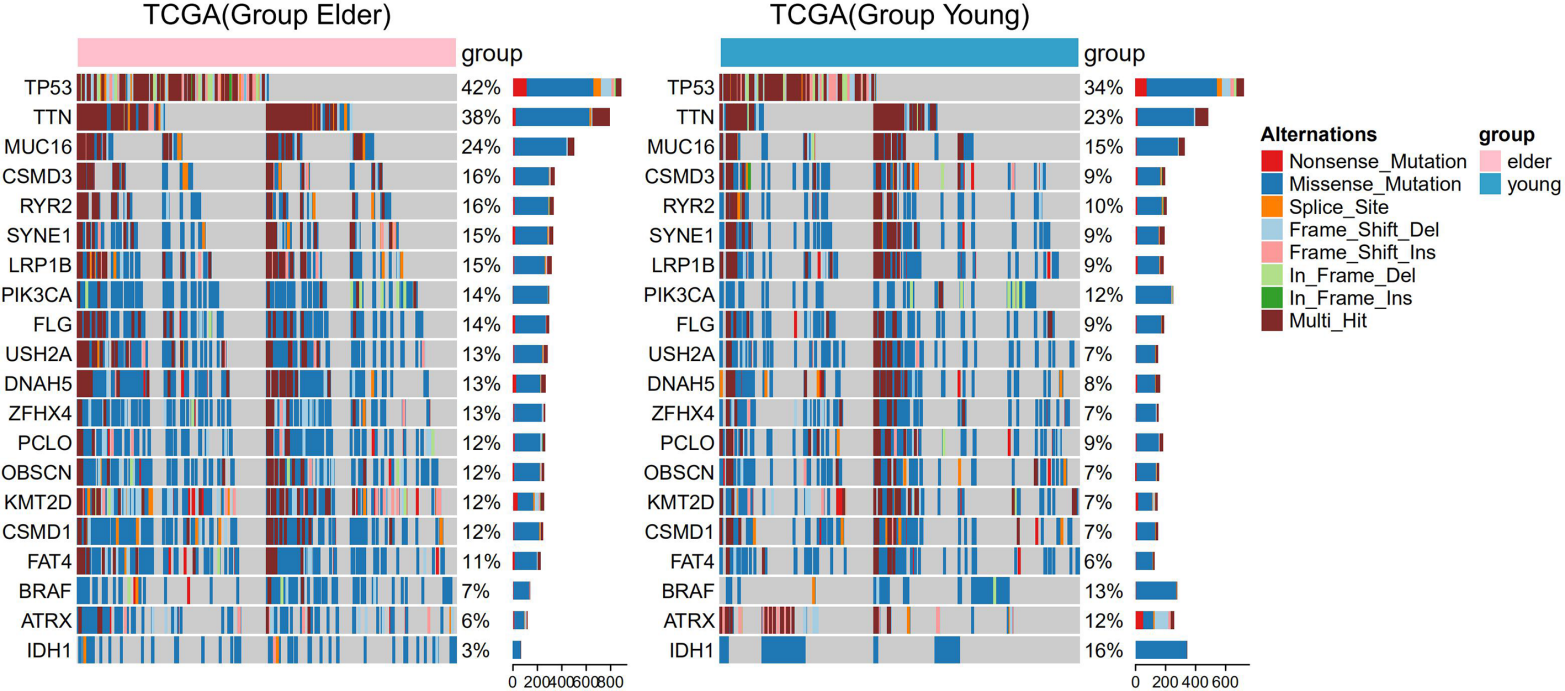
Panoramic images of gene mutations in the elderly group (≥ 50 years old) and the young group of patients in The Cancer Genome Atlas (TCGA) pan-cancer dataset. The bar graph on the right shows the mutation frequency of each gene.

There were 166 significantly different mutant genes in the TCGA database between the elderly group and the young group. Except for IDH1 mutation, BRAF mutation and ATRX mutation, the mutation frequencies of other genes (such as TP53, TTN, MUC16, and LRP1B) were higher in the elderly group. We selected 20 of the 166 genes for visualization, including 3 genes (IDH1, BRAF and ATRX) with a higher mutation frequencies in the young group and 17 genes with a mutation frequency of more than 10% in the elderly group (**Figure 3**). In addition, through the analysis of TCGA gene expression, we found that compared with those in the young group, some genes were significantly upregulated or downregulated in the elderly group, as shown in the volcano plot (**Figure S4**).

### Analysis of TMB, NAL, and DDR Pathway Mutations Between the Elderly Group and the Young Group

**Figure 4:**
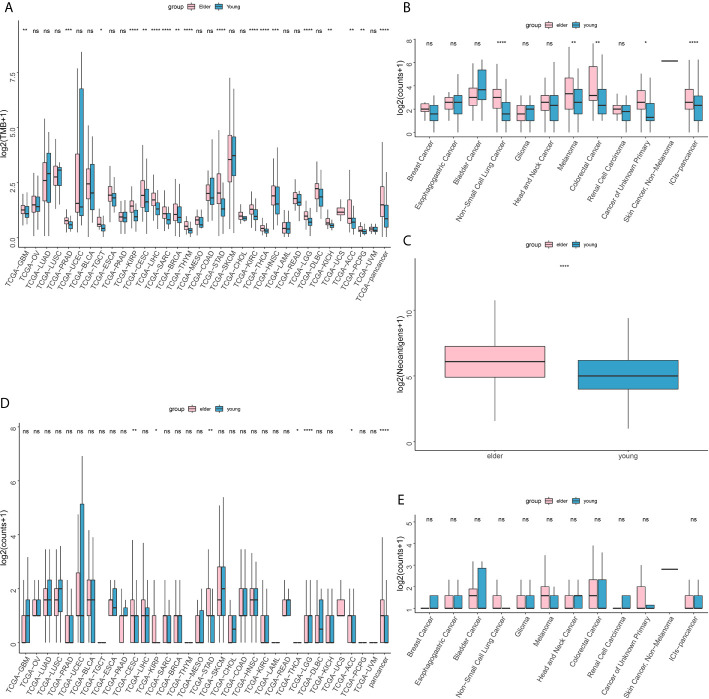
**(A)** Difference in tumor mutation burden (TMB) between the older (≥ 50 years old) and younger groups in The Cancer Genome Atlas (TCGA) database. **(B)** Difference in tumor mutation burden (TMB) between the older (≥ 50 years old) and younger groups in the Memorial Sloan-Kettering Cancer Center (MSKCC) immunotherapy cohort. **(C)** Difference in neoantigen load (NAL) between the older (≥ 50 years old) and younger groups in The Cancer Genome Atlas (TCGA) database. **(D)** Difference in DDR pathway mutations between the older (≥ 50 years old) and younger groups in The Cancer Genome Atlas (TCGA) database. **(E)** Difference in DNA damage response and repair (DDR) pathway mutations between the older (≥ 50 years old) and younger groups in the Memorial Sloan-Kettering Cancer Center (MSKCC) immunotherapy cohort. ns, no significant difference *P < 0.05, **P < 0.01, ***P < 0.001, ****P < 0.0001.

TCGA database analysis showed that in pan-cancer patients and in patients with most cancer types, the TMB of elderly patients was higher than that of young patients (**Figure 4A**). The results of MSKCC immunotherapy cohort analysis showed that for pan-cancer patients treated with ICIs, the TMB of elderly patients was higher than that of young patients (**Figure 4B**). The NAL was elevated in the elderly group compared with the young group (**Figure 4C**).

In addition, in the TCGA database analysis, the DDR pathway mutation rate in the elderly group was higher than that in the young group (**Figure 4D**). In the MSKCC immunotherapy cohort, however, there was no significant difference in DDR mutation rate between the elderly group and the young group (**Figure 4E**).

### Analysis of the Expression of Immune-Related Genes and Immune Cell Infiltration Between the Elderly Group and the Young Group

**Figure 5:**
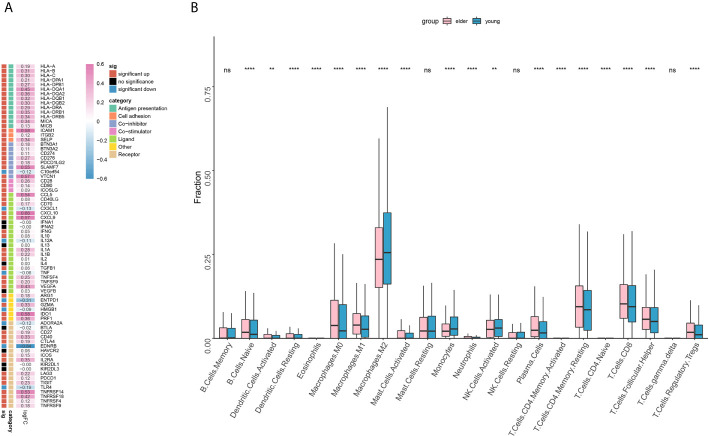
**(A)** The expression of immune-related genes in the elderly group (≥ 50 years old) compared with the young group. Statistical significance and categories of immune-related genes are shown in the bar on the right. **(B)** Differences in the infiltration scores of 22 immune cell types between the older (≥ 50 years old) and younger groups. ns, no significant difference. **P < 0.01, ****P < 0.0001.

Through the analysis of the expression of immune-related genes, we found that most of the immune-related genes were significantly highly expressed (**Figure 5A**) in the elderly group. For example, the expression of antigen presentation-related genes, cell adhesion-related genes and costimulatory factor-related genes was significantly increased in the elderly group. Then, we analyzed the infiltration scores of 22 kinds of immune cells, and the results showed that among most kinds of immune cells, the infiltration scores in the elderly group were higher than those in the young group (**Figure 5B**). It is worth noting that the infiltration of CD8+ T cells, M1 macrophages and regulatory T cells (Tregs) was significantly increased in the elderly group, while that of M2 macrophages was significantly increased in the young group.

### Analysis of the Correlation Between the Infiltration Score of Immune Cells and the Enrichment Score of the GSVA Pathway

**Figure 6:**
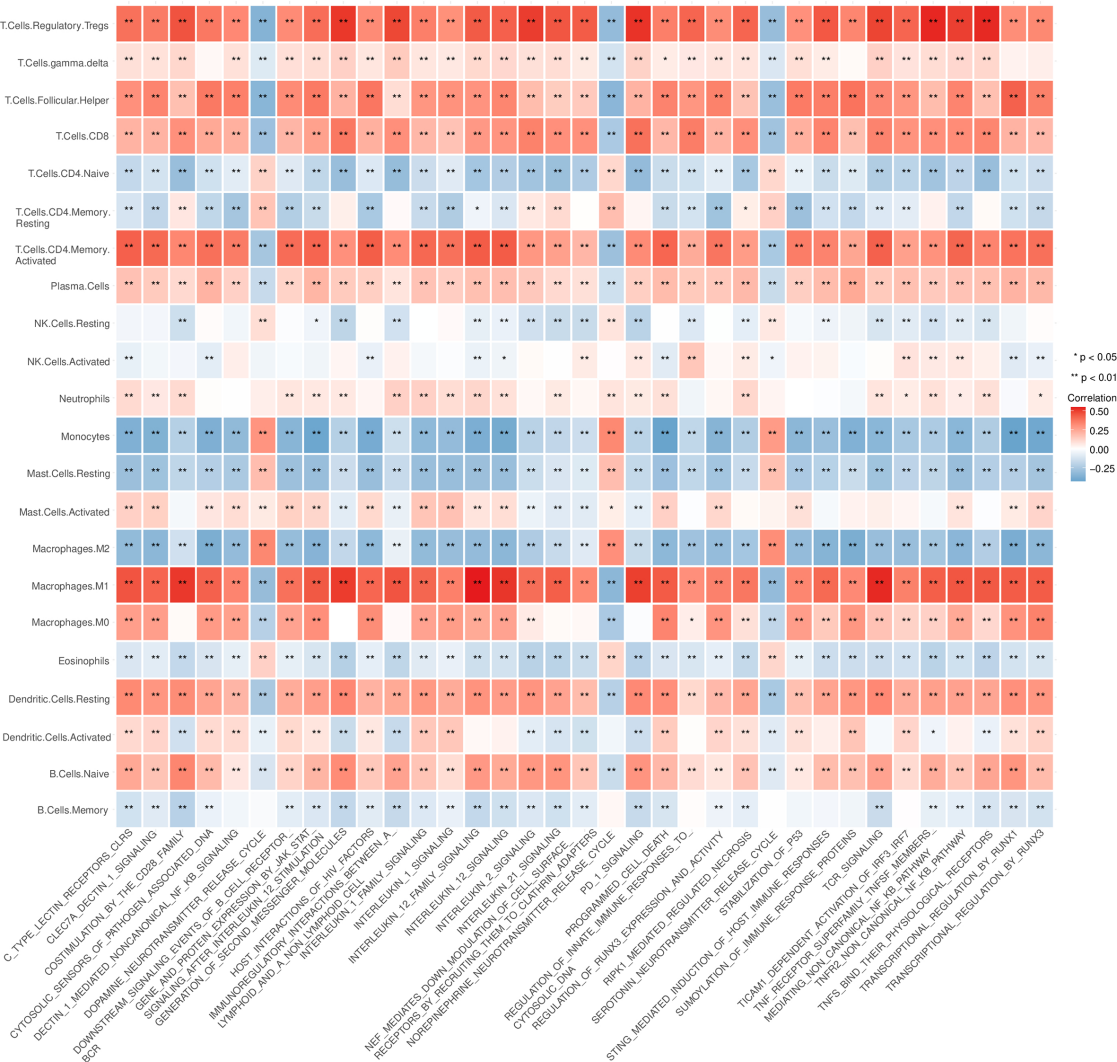
4Analysis of the correlation between the infiltration score of immune cells and the pathway enrichment score of the gene set variation analysis (GSVA). *P < 0.05, **P < 0.01.

In the analysis of the differences in GSVA pathways, 965 differentially expressed pathways were obtained by setting the threshold to adj.p.value < 0.01. The correlations between these pathways and the infiltration scores for 22 kinds of immune cells (obtained by CIBERSORT analysis) were analyzed, and the pathways highly associated with macrophage, Treg and CD8+ T cell infiltration were screened out for visualization (**Figure 6**).

## Discussion

ICIs are effective against various cancers (27), lack known cumulative toxicity and are safer than conventional cytotoxic chemotherapies (15). Therefore, ICIs represent an attractive choice for the treatment of elderly patients. In previous clinical studies, the ages of 65 or 70 years are often used to distinguish between the elderly and the young. However, any certain age cutoff is insufficient for characterizing elderly individuals because aging is a highly variable physiological process (28). To reduce the possible deviation caused by a specific age limit, we set a number of different age cutoff points (50, 55, 60, 65, 70, and 75) and consequently observed the differences in survival outcomes brought about by different cutoff points. Our results showed that the 50 years old is a good age cutoff point for prognosis. Therefore, using the data in the database and literature, we divided people into the elderly group and the young group according to the cutoff of 50 years old and further explored the biomarkers related to age.

In this study, we found that TMB, NAL and the number of DDR pathway mutations in the elderly group were significantly higher than those in the young group of pan-cancer patients. TMB refers to the total number of nonsynonymous mutations present in tumors (20), which produce abnormal proteins by altering amino acid sequences. If abnormal proteins are eventually recognized by immune cells, they are likely to become neoantigens that facilitate the subsequent immune response (29). High-TMB tumors tend to generate more neoantigens and are more immunogenic, thus responding better to ICIs (30). Samstein et al. included a large cohort of 1662 cancer patients receiving ICIs and demonstrated that high TMB can predict better OS across multiple cancer types, such as colorectal cancer, NSCLC, and head and neck cancer (18). Another comprehensive analysis suggested that for patients treated with ICIs in the pan-cancer dataset, high TMB was significantly related to favorable OS (HR=0.40; 95% CI: 0.30-0.53; p<0.00001) and a low risk of disease progression (HR=0.37; 95% CI: 0.26-0.53; p<0.00001) (31). Subgroup analysis showed that the prognostic effect of TMB was independent of TMB detection method and tumor type (31). A number of clinical trials, such as Keynote-001, also showed a positive correlation between TMB and ICI response (32). Other studies have shown that the number of mutations targeted by T cells, namely, the true NAL, may be more associated with the ICI response than TMB (33). In addition, DDR mutations usually lead to DNA damage and repair defects, resulting in genomic instability and TMB increase, and may enhance the antitumor immune response through neoantigen-dependent and neoantigen-independent mechanisms (34, 35). Several studies have revealed that DDR mutations are related to better prognosis of patients receiving ICIs (36, 37). In conclusion, the increase in efficacy of ICIs in the elderly group relative to that of the young group may be attributed to an increase in TMB, NAL and DDR mutations in the elderly group.

From the perspective of gene mutations, our results suggested that there were 166 significantly different genes between the elderly group and the young group, and almost all of genes had higher mutation frequencies in the elderly group. TP53 was the most common mutation in both the elderly and young groups. Studies have shown that the correlation between TP53 mutation and tumor immunity is related to the cancer type. For example, TP53-mutated lung adenocarcinoma and breast cancer exhibit enhanced PD-L1 expression and immune cell infiltration, which indicate a good response to ICIs, while in colon adenocarcinoma, head and neck squamous cell carcinoma and stomach adenocarcinoma, TP53 mutation showed the opposite effect (38). This may be explained by the fact that TP53 mutation can lead to chromosome/genomic instability, thus increasing TMB and tumor aneuploidy level (TAL) (39). Since TMB and TAL are positively and negatively correlated with the immunotherapy response, respectively, the therapeutic response of TP53 mutant cancer to ICIs may be influenced by both the TMB and TAL (40). Chen et al. analyzed the immunotherapy cohort of melanoma and NSCLC patients and found that the LRP1B-mutant group had higher TMB and NAL and better prognosis than the wild-type group (41). This may be attributed to the characteristics of LRP1B mutant tumors, that is, the abundance of genes involved in antigen processing and presentation and cell cycle checkpoints (41). In the 2020 American Society of Clinical Oncology (ASCO), a multicenter study including an ICI cohort of different cancer species showed that the ORR in patients with LRP1B mutations was significantly higher than that in wild-type patients (57% *vs* 18%), independent of TMB/microsatellite instability (MSI) status. Moreover, LRP1B mutations were associated with longer OS (HR= 0.58, 95% CI: 0.36-0.95) and PFS (HR=0.39, 95% CI: 0.24-0.63) (7). As a result, LRP1B may be an independent prognostic indicator for predicting the efficacy of ICIs. In addition, studies have reported that in the pan-cancer immunotherapy dataset, MUC16 and TTN mutations were associated with higher TMB and better OS (42). Using the TIDE algorithm, Li et al. found that the SYNE1-mutant group had a stronger response to ICI treatment than the wild-type group (43). In tumors with RYR2 or CSMD3 mutations, the CXCL9 expression level is elevated, which can promote T cell migration and activation and facilitate the antitumor immune response (44, 45). In summary, the specific gene mutations mentioned above may explain the better prognosis of patients aged ≥50. It is worth noting that considering the dual role of TP53 mutation, TP53 mutation may result in different outcomes in pan-cancer datasets consisting of different cancer types or frequencies.

The frequency of mutations in some genes was higher in the younger group; for example, the BRAF mutation, which mainly occurs in melanoma and is conducive to the generation of an immunosuppressive microenvironment, was more common in the younger group. It has been reported that BRAF mutation could inactivate the antitumor immune response by upregulating the MAPK signaling pathway in melanoma (46). BRAF-mutant melanoma may achieve immune escape through several mechanisms, including preventing antigen-presenting cells from presenting tumor antigens and subsequently activating T cells, resulting in the low expression of human leucocyte antigen (HLA) class I molecules and melanoma differentiation antigens, and promoting the accumulation of myeloid-derived suppressor cells and regulatory T cells (47–49). In conclusion, BRAF mutation appears to be detrimental to patients’ response to ICIs, which is consistent with the trend of our findings. Since BRAF inhibitors may induce increased PD-L1 expression in tumor cells and help restore the immune-stimulating microenvironment of BRAF-mutant melanoma (50), the combination of BRAF inhibitors and ICIs may provide a stronger antitumor effect.

Next, we analyzed the difference in the expression of 75 immune-related genes between the elderly group and the young group. The results showed that the immune genes related to antigen presentation, such as HLA-I (HLA-A, HLA-B and HLA-C) and HLA-II (HLA-DR, HLA-DQ and HLA-DP), which are responsible for presenting antigens to CD8+ T cells and CD4+ T cells, respectively, were highly expressed in the elderly group (51). The interaction between MICA/B and NKG2D activates the cytotoxicity of natural killer (NK) cells, which has been reported as an important costimulation signal of T cells (52). The expression of cell adhesion-related genes was significantly increased in the elderly group. As one of the cell adhesion molecules, ICAM-1 can bind to LFA-1 on CD8+ T cells and promote the activation and cytotoxicity of CD8+ T cells in a TCR-dependent manner (53). Consistent with this, the expression of ICAM-1 is negatively correlated with the incidence of lymph node or distant metastasis in patients with breast cancer and colorectal cancer (54, 55), suggesting a favorable prognosis. However, it has also been reported that the expression of ICAM-1 is positively correlated with the metastatic potential of some tumors (56). The mechanism by which ICAM-1 plays a dual role in the development of tumors is still unclear, and the relationship between ICAM-1 and the therapeutic response of ICIs remains to be further explored. Immune genes associated with chemokines, such as CCL5, CXCL9 and CXCL10, were highly expressed in the elderly group. Increased levels of CXCL9 and CXCL10 have been reported to be related to elevated tumor CD8+ T cell density and improved survival in patients with a variety of cancers (57, 58). CXCR3 is the coreceptor of CXCL9/10/11. In the tumor microenvironment, the CXCL9/10/11-CXCR3 signaling pathway can exert antitumor immunity through multiple mechanisms (i.e., promoting the chemotaxis movement of CXCR3-activated immune cells to tumor sites (59) and activating the STAT and PI3K-Akt signaling pathways, thus upregulating PD-L1 expression, which usually means a good response to ICIs) (60). It is well known that the sustained antitumor effect of ICIs is closely related to high expression levels of immune checkpoint molecules. In line with this, our results suggested that the expression of immune checkpoint-related genes, such as PDCD1 [also known as PD1/CD279; ligand: PDL1, PDL2 (PDCD1LG2)], CD274 (also known as PDL1; ligand: PD1), CTLA4 (also known as CD152, ligand: CD80, CD86), LAG3 (ligand: MHC-II) and TIGIT (ligand: CD155, CD112, CD113), was higher in the elderly group. In addition, LAG3 and TIGIT are usually coexpressed and upregulated along with PD1 on tumor-infiltrating lymphocytes (TILs) (61).

After analyzing the degree of immune cell infiltration, we found that most of the immune cell subtypes, such as CD8+ T lymphocytes, Tregs and M1 macrophages, were highly infiltrated in the elderly group, while M2 macrophages were highly infiltrated in the young group. TILs are a predictive biomarker of response to ICI treatment. The greater the number of TILs, the stronger the patients’ response to ICIs (62). Among TILs, CD8+ T cells are the immune cells that have the strongest positive effect on the survival of cancer patients. Bruni et al. confirmed the positive prognostic value of CD8+ T cells in 18700 patients across 17 cancer types (63). As a subtype of CD4+ T cells, Tregs suppress the antitumor immune response and enhance the immune escape of tumor cells. To date, the association between Tregs and poor prognosis in renal and cervical cancer has been well established (64, 65), while the positive impact of Tregs on survival has been demonstrated in bladder cancer, hematological malignancies and head and neck cancer (66–68). The controversial result may contribute to the fact that the definition of Tregs is inconsistent in previous studies, most of which only rely on FOXP3 as a marker for Tregs (63). The dual role of macrophages in the process of tumor development is related to their polarization state. For example, M1 macrophages release IL-1 and IL-12 under stimulation by factors such as interferon-γ and tumor necrosis factor-α to play proinflammatory and antitumoral roles (69). In contrast, under the stimulation of IL-4 and IL-13, M2 macrophages secrete mediators, such as IL-10 and transforming growth factor β, that contribute to the establishment of a tolerable microenvironment to exert anti-inflammatory and protumoral effects (69). Through experimental research, Du et al. found that exosomes derived from M1 macrophages can directly regulate T cells, promoting Th1 cell differentiation (increasing the proportion) and effector functions (increasing IFN-γ intensity) and increasing the production of IFN-γ by CD8+ T cells (70). Moniek’s data on the existence, induction, and plasticity of antigen-presenting cells in cervical cancer indicated that tumor-infiltrating Th1 cells could stimulate a tumor-rejecting environment by converting M2 macrophages to M1 macrophages (71). Thus, the better prognosis of elderly cancer patients receiving ICI therapy may be closely related to the type and degree of immune cell infiltration in the immune microenvironment.

By analyzing immune cell-related pathways, we found that the TCR signaling pathway, CD28 costimulation signaling pathway, IL-2 signaling pathway, IL-12 signaling pathway, PD1 signaling pathway and NF-κB signaling pathway were significantly positively correlated with CD8+ T lymphocytes, Tregs and M1 macrophages and significantly negatively correlated with M2 macrophages. It is well known that the effective activation of T cells depends on the recognition of MHC-binding antigenic peptides by TCRs and the role of costimulatory signals. Subsequently, activated T cells promote IL-2 signaling, which promotes the proliferation, differentiation and survival of T lymphocytes, as well as the killing activity of NK cells (72). NF-κB can be activated either by the classical pathway under a variety of inflammatory signals, such as proinflammatory cytokines and Toll-like receptors, or by the nonclassical pathway, which is induced by ligands from the tumor necrosis factor receptor family (73). It has been reported that NF-κB can induce the production of chemokines and cytokines, attract immune cells, and maintain a proinflammatory tumor immune microenvironment (74, 75). At the same time, the NF-κB signaling pathway is also involved in the regulation of tumor cell immune checkpoint expression, which can induce PD-L1 expression, indicating a good response to ICIs (76, 77).

The advantages of this study are listed as follows. We tried to explore the influence of different age cutoff points on the prognosis of cancer patients treated with ICIs. According to the results, a suitable cutoff point of 50 years old was selected, and patients were grouped accordingly. In addition, we used the database and data from related literature to try to reveal the biological factors related to age. The limitations of this paper are listed as follows. Due to limited data sources, it is not clear whether patients in the MSKCC clinical cohort received other treatments before ICIs. In addition, our exploration of genetic and other biological factors lacks direct prognostic data support.

## Conclusion

Cancer patients aged ≥50 years can benefit more from ICIs than younger patients. This may be related to specific gene mutations, gene expression levels and the degree of immune cell infiltration related to age. In the future, prognostic data are still needed to directly verify the underlying mechanisms behind the age cutoff point of 50 years.

## Data Availability Statement

The datasets presented in this study can be found in online repositories. The names of the repository/repositories and accession number(s) can be found below: Mutations-mc3.v0.2.8.PUBLIC.maf. (https://gdc.cancer.gov/about-data/publications/pancanatlas), RNA (Final) -EBPlusPlusAdjustPANCAN_IlluminaHiSeq_RNASeqV2.geneExp.tsv (https://gdc.cancer.gov/about-data/publications/pancanatlas), https://www.cbioportal.org/study?id=tmb_mskcc_2018.

## Author Contributions

Study concept and design: JZ and PL. Data analysis: RG, QL, AL, and JL. Drafting of the manuscript: RG. Critical revision of the manuscript for important intellectual content: WD and MC. All authors contributed to the article and approved the submitted version.

## Funding

This study was supported by grant from Natural Science Foundation of Guangdong Province (2018A030313846).

## Conflict of Interest

The authors declare that the research was conducted in the absence of any commercial or financial relationships that could be construed as a potential conflict of interest.

## Supplementary Material

Supplementary Figure 1 Kaplan-Meier curves depicting overall survival (OS; in months) according to different age cutoff points in patients receiving PD-1/PD-L1 inhibitors (the PD-1/PD-L1 group).

Supplementary Figure 2 Kaplan-Meier curves depicting overall survival (OS; in months) according to different age cutoff points in patients receiving CTLA-4 inhibitors (the CTLA-4 group).

Supplementary Figure 3 Kaplan-Meier curves depicting overall survival (OS; in months) according to different age cutoff points in patients receiving a combination of PD-1/PD-L1 inhibitors and CTLA4 inhibitors (the combo group).

Supplementary Figure 4 Differences in gene expression between the elderly group (≥50 years old) and the young group of pan-cancer patients in The Cancer Genome Atlas (TCGA) database. We annotated genes with log fold change (FC) > 1.5.
