# Supplementary figures and images for: Influence of Different Age Cutoff Points on the Prediction of Prognosis of Cancer Patients Receiving ICIs and Potential Mechanistic Exploration
Source: Front Oncol. 2021 Jun 23;11:670927. doi: 10.3389/fonc.2021.670927 (PMC8260982; doi:10.3389/fonc.2021.670927)

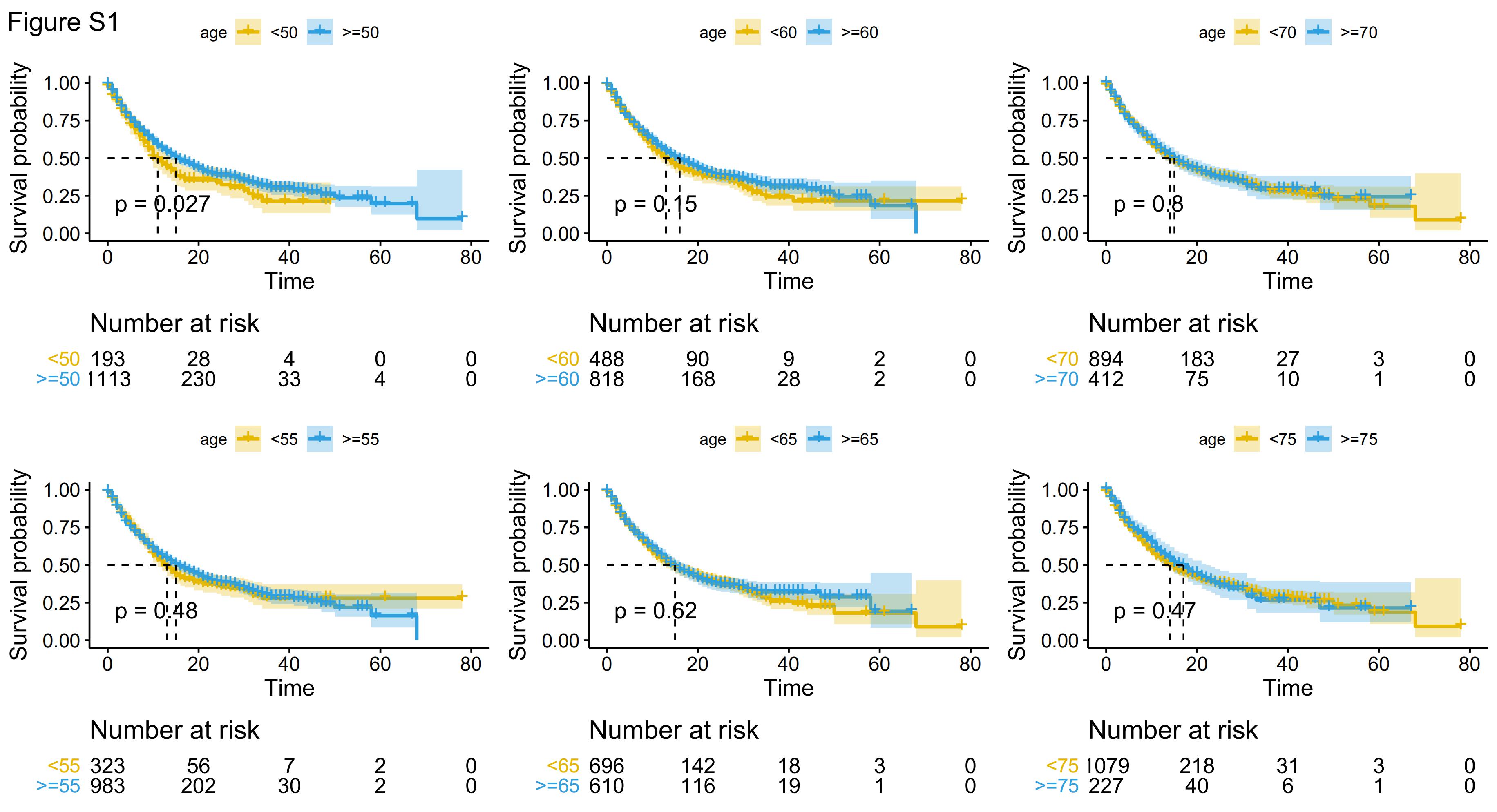

Supplement: Supplementary Figure 1 — Kaplan-Meier curves depicting overall survival (OS; in months) according to different age cutoff points in patients receiving PD-1/PD-L1 inhibitors (the PD-1/PD-L1 group). [file Image_1.jpeg]

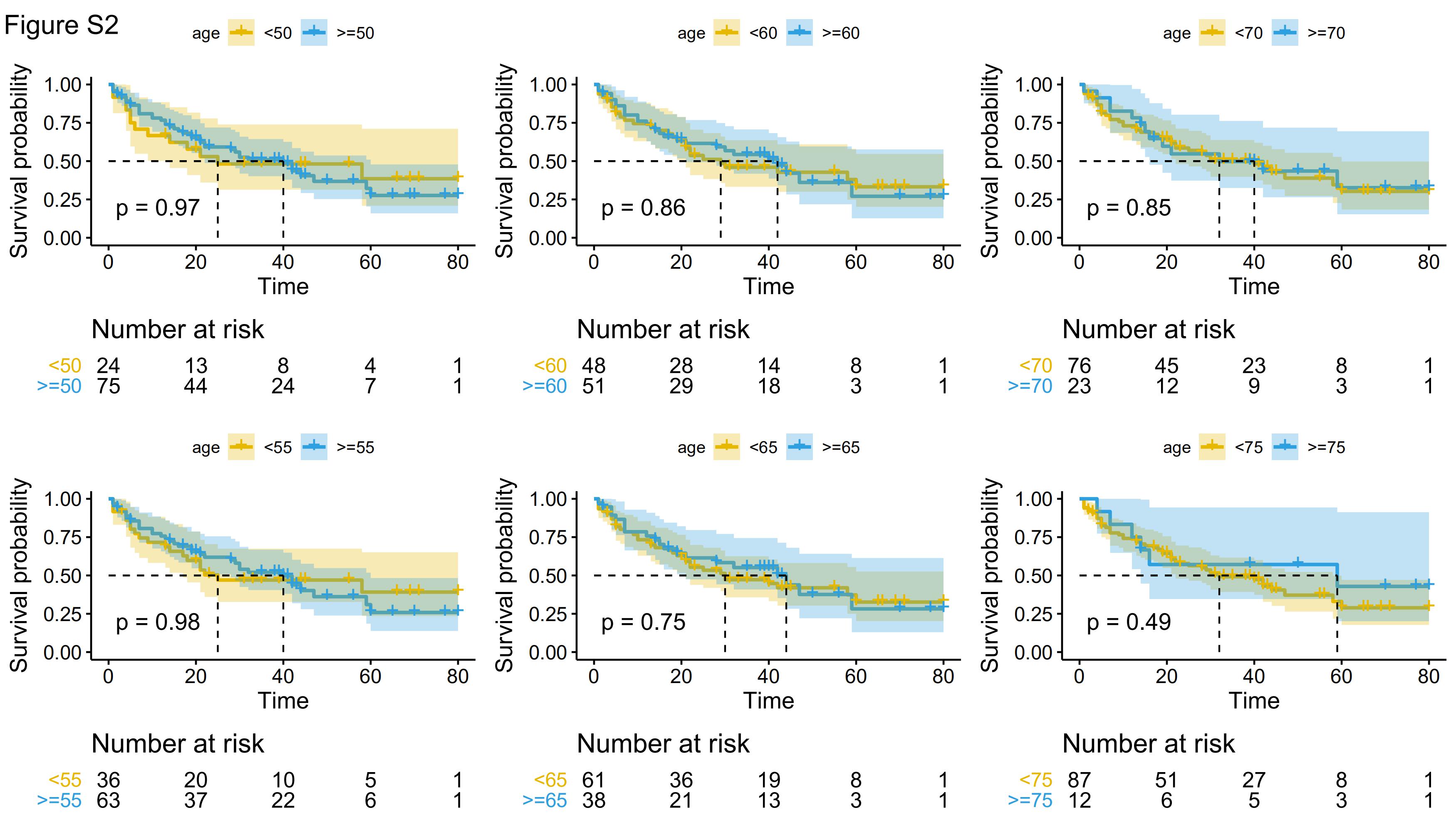

Supplement: Supplementary Figure 2 — Kaplan-Meier curves depicting overall survival (OS; in months) according to different age cutoff points in patients receiving CTLA-4 inhibitors (the CTLA-4 group). [file Image_2.jpeg]

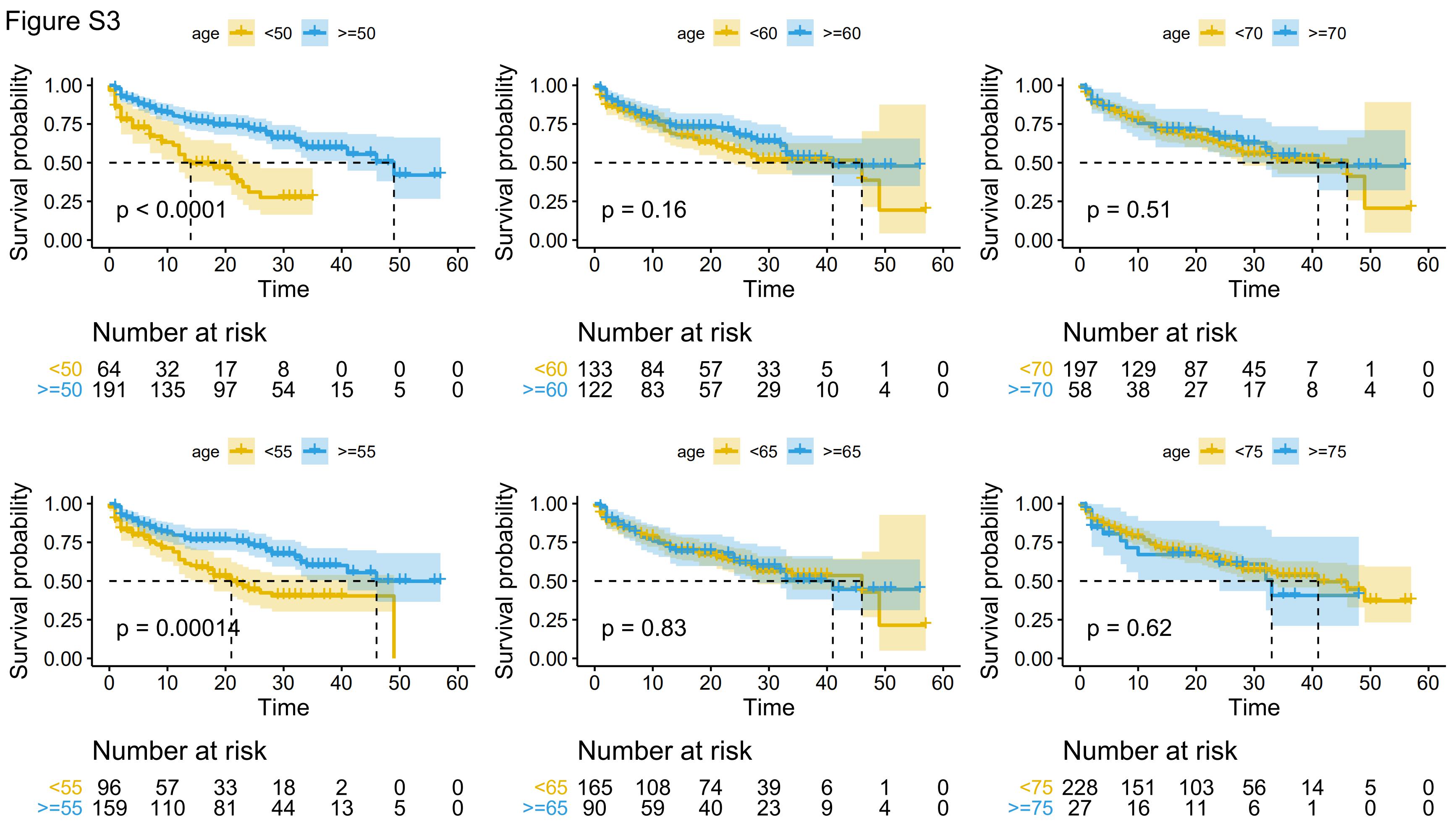

Supplement: Supplementary Figure 3 — Kaplan-Meier curves depicting overall survival (OS; in months) according to different age cutoff points in patients receiving a combination of PD-1/PD-L1 inhibitors and CTLA4 inhibitors (the combo group). [file Image_3.jpeg]

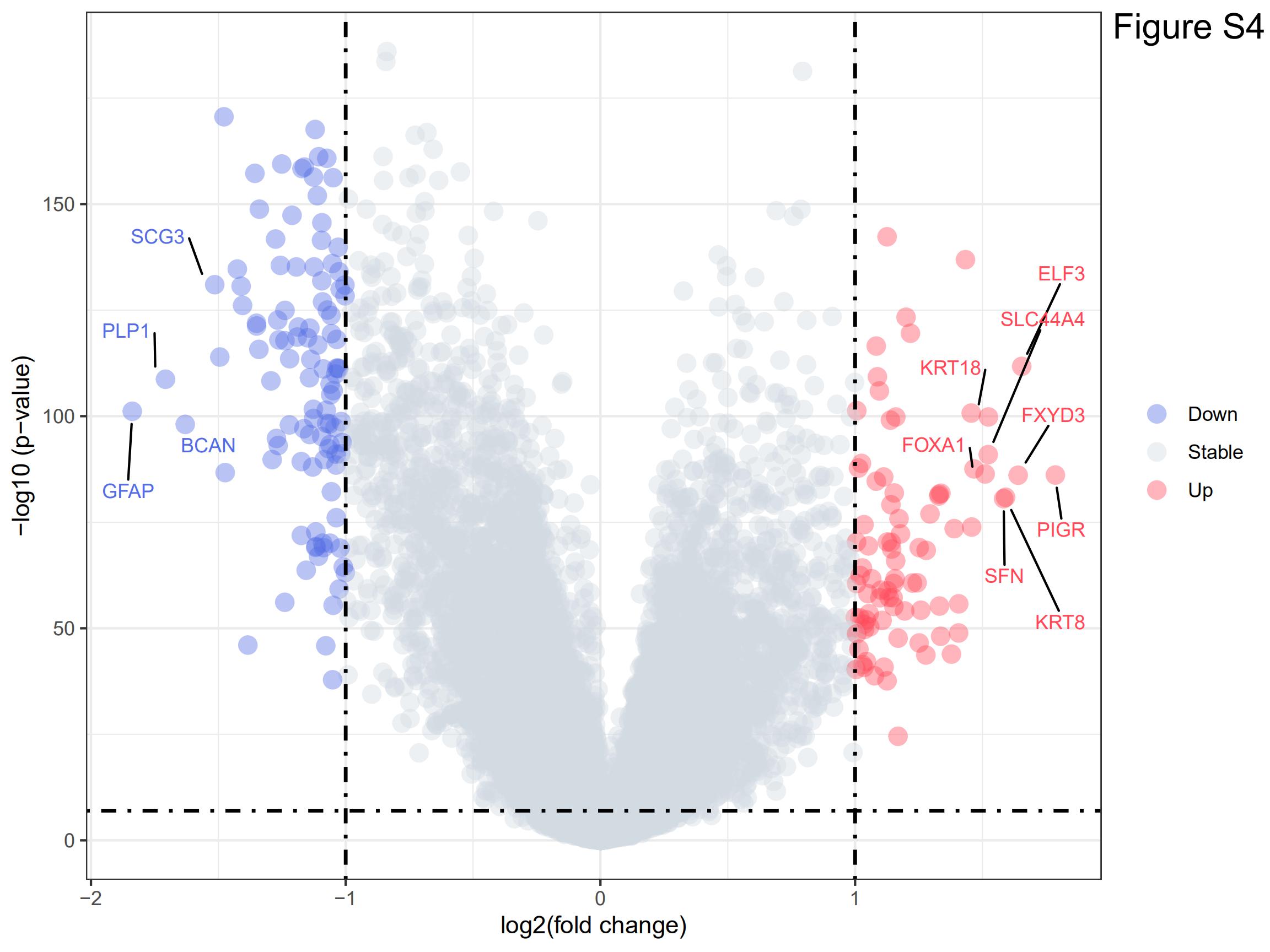

Supplement: Supplementary Figure 4 — Differences in gene expression between the elderly group (≥50 years old) and the young group of pan-cancer patients in The Cancer Genome Atlas (TCGA) database. We annotated genes with log fold change (FC) > 1.5. [file Image_4.jpeg]
